# Associations between dietary patterns and adequate intake of climate-sensitive micronutrients among young children living in Siaya County, Kenya: Findings from the ALIMUS study baseline data

**DOI:** 10.1186/s41043-026-01293-y

**Published:** 2026-03-26

**Authors:** Grace Wothaya Kihagi, Isabel Mank, Adi Lukas Kurniawan, Tracy Bonsu Osei, Gabriele Stieglbauer, Michael Mbata, Erick M. O. Muok, Raissa Sorgho, Ina Danquah

**Affiliations:** 1 Transdisciplinary Research Area Technology and Innovation for Sustainable Futures and Center for Development Research (ZEF), Rheinische Friedrich-Wilhelms University of Bonn, Bonn, Germany; 2 Heidelberg Institute of Global Health (HIGH), Medical Faculty and University Hospital, Heidelberg University, Heidelberg, Germany; 3 German Institute for Development Evaluation (DEval), Bonn, Germany; 4 Center for Global Health Research (CGHR), Kenya Medical Research Institute (KEMRI), Kisumu, Kenya; 5 Public Health Institute (PHI), Center for Wellness and Nutrition (CWN), Sacramento, CA USA

**Keywords:** Dietary patterns, Children, Sub-Saharan Africa, AFPQ, Climate change, Micronutrient adequacy

## Abstract

**Background:**

Climate change impacts food security in sub-Saharan Africa due to harvest losses and micronutrient reductions in staple foods. We aimed at identifying dietary patterns and their associations with climate-sensitive micronutrients (iron, zinc, selenium, vitamin A) among young children in rural Kenya.

**Methods:**

We analyzed baseline data (*N* = 626; age range: 6–23 months, male sex: 54%) of a cluster-randomized controlled trial for nutrition interventions in Siaya county. Nutrient adequacy ratios (NARs) were calculated from semi-quantitative food frequency data. Dietary patterns were derived from food groups by Principal Component Analysis. In multiple-adjusted logistic regression models, we calculated odds ratios (OR) and 95% confidence intervals (CIs) for the associations between dietary patterns and climate-sensitive micronutrients.

**Results:**

The proportions of micronutrient inadequacy (NAR < 80%) were 85% for iron, 63% for zinc, 31% for Vitamin A, and 11% for selenium. Pattern 1 explained 12.7% of the variation in food intake and was inversely associated with iron inadequacy (OR: 0.20; 95% CI: 0.11, 0.37) and zinc inadequacy (OR: 0.57; 95% CI: 0.38, 0.86). Pattern 2 explained 8.0% and was positively associated with iron inadequacy (OR: 1.54; 95% CI: 1.10, 2.16). Pattern 3 explained 6.4% and showed higher odds of zinc inadequacy (OR: 1.87; 95% CI: 1.43, 2.44) and selenium inadequacy (OR: 1.95; 95% CI: 1.25, 3.06) in the fully adjusted models.

**Conclusions:**

The intakes of iron, zinc, and vitamin A remain inadequate among young children in Siaya county. Pattern 1, but not the modernized diets (patterns 2 and 3), appears to support adequate intake of climate-sensitive micronutrients. Nutrition-specific and nutrition-sensitive programs are recommended to strengthen micronutrient adequacy in this region.

**Trial registration:**

German Clinical Trials Register (DRKS) DRKS00019076.

**Graphical abstract:**

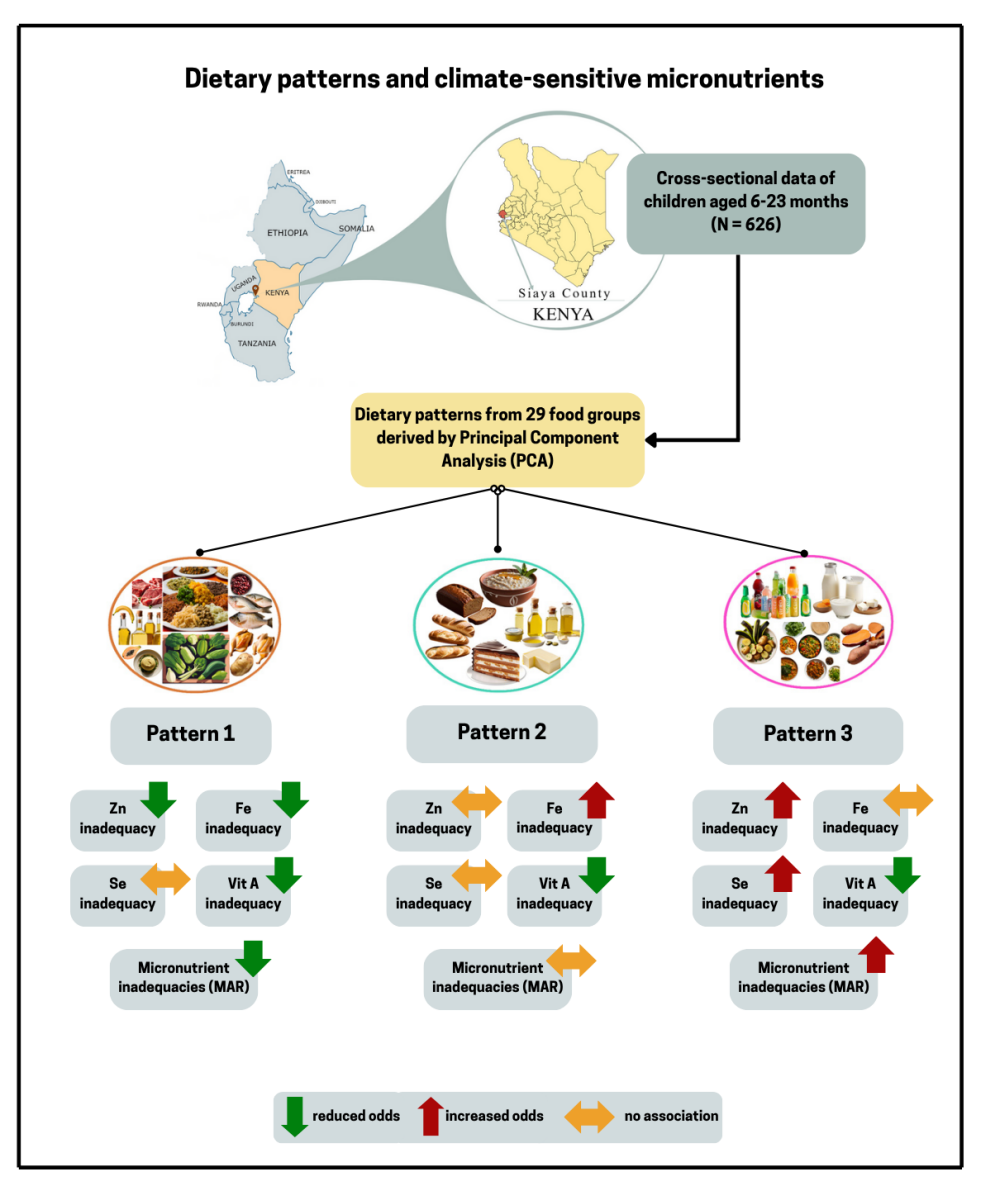

**Supplementary Information:**

The online version contains supplementary material available at 10.1186/s41043-026-01293-y.

## Background

Young children in sub-Saharan Africa (SSA) are at risk of insufficient micronutrient intakes, which can increase susceptibility to infectious diseases and other illnesses and may reduce their overall survival rates [1]. Deficiencies of micronutrients, including iron, zinc, and vitamin A, are highly prevalent in young children in SSA [2, 3]. Recent data from Kenya highlight that on average 83% of children aged 6–59 months have low plasma zinc (< 0.1 µmol/L), 22% face iron deficiency (ferritin < 12 µg/L), and 9% have vitamin A deficiency (retinol-binding protein < 0.7 µmol/L) [4]. Yet these numbers differ according to region, socio-economic class, and infectious disease status [4].

Climate change impacts on food systems through direct and indirect pathways, which aggravate inadequate intakes of important micronutrients [5, 6]. Quantitatively, weather extremes and fluctuations in rainfall, which are components of a changing climate, can result in crop losses and decreased household food production [7, 8]. In 2022, for instance, reductions in major staples (maize) were attributed to reduced rainfall across the seasons. In 2023, favourable weather was associated with a shift to increased maize production by 38.8% [9]. Qualitatively, increased levels of atmospheric CO_2_ have been shown to reduce selenium, iron, and vitamin A in major food crops, including rice, sorghum, and maize [10], with potential losses of zinc (Zn) by 1–9%, iron (Fe) by 4–5% [11, 12], vitamin A by 15% [13, 14], and selenium (Se) by 9% [15]. While there is no recent field data from Kenya itself, mechanistic studies, including Free-Air CO_2_ Enrichment (FACE) and Open-Top-Chamber (OTC) experiments, confirm the reduction of micronutrients upon increased atmospheric CO_2_ concentrations in major food crops relevant for Kenyan smallholder subsistence farmers [16]. Besides, studies conducted on the nexus between climate change and food security in SSA have attributed a 1.2%-decrease in food security (= index of food availability, access, utilization, and stability) to a 1.2%-rise in carbon emissions [17]. Therefore, with the projected rise in global temperatures, including the rising trend in Kenya, as well as the observed and projected micronutrient losses in major food crops, young children in Kenya may be at risk of inadequate intakes of iron, zinc, selenium, and vitamin A. Hence, we refer to these nutrients as ‘climate-sensitive micronutrients’.

On the background of current trends in child nutritional status in Kenya, appropriate food consumption and feeding practices are essential to achieve adequate nutrient intakes [1]. Indeed, only one-third of children aged 6–23 months in Kenya meet the criteria for a Minimum Acceptable Diet (MAD) [18]. Nutrition education and counselling, among others, are useful strategies to equip households with such knowledge and practice to strengthen food security and thus, adequate nutrient intakes for children [10, 19, 20]. To provide appropriate and context-specific nutrition education, it is essential to understand the complexity of food consumption in young children and its association with adequate intakes of climate-sensitive micronutrients. Therefore, this study aimed to identify and describe exploratory dietary patterns and determine their associations with adequate intakes of climate-sensitive micronutrients among young children living in rural Kenya.

## Methods

### Study design and setting

This study used a cross-sectional dataset of a cluster-randomized controlled trial, called “*ALIMUS” (Latin: We are feeding*!) [21], which was nested in the existing Health and Demographic Surveillance System (HDSS). The trial evaluates an integrated intervention involving home gardening and behaviour change communication that aims at reducing undernutrition in young children of Siaya county. The setting often experiences high temperatures, and the population shows unique vulnerabilities like high malaria prevalence, HIV, and other infectious diseases [21]. The analysis and reporting of the baseline data adhered to the STROBE (Strengthening the Reporting of Observational Studies in Epidemiology) guidelines for reporting findings from cross-sectional studies [22], and the corresponding checklist is presented in Supplementary Table 1. Baseline data of children aged 6–23 months living in Siaya County were collected between July and December 2021. Siaya County experiences two rainy seasons: long rains between March and May, and short rains between October and December, with rainfall ranging from 1200 to 2000 mm. The temperature ranges between 21^°^ and 24^°^C yearly [23]. The population of Siaya county, Western Kenya, relies mainly on subsistence farming and fishing to meet their nutritional and economic needs. The primary food crops produced are maize, beans, sweet potatoes, sorghum, rice, and cassava. In this region, few legumes and starchy staples are the main components of children’s diets [21]. Research on dietary patterns among young children in Kenya is lacking. The data for school-aged children in Western Kenya demonstrates their low dietary diversity and inadequate micronutrient intakes [24].

## Sampling and recruitment

Sampling and recruitment were conducted at the household level, targeting households with children in the complementary feeding period (6–23 months). The sample size for the ALIMUS trial was based on the primary outcome of the intervention (HAZ), which yielded a total of 600 children [21]. For the post hoc sample size calculation, we assumed an alpha level of 0.05, 80% power, and the observed odds ratio for Fe inadequacy of 0.20. The corresponding sample size is 64 for each group increase in the exposure variable. Given that dietary pattern scores have a mean of zero and a standard deviation of 1, each score point increase reflects one standard deviation. The maximum number of units, therefore, ranges from − 3 SD to + 3 SD, resulting in 6 × 64 participants for the present analysis. This means, a sample size of 384 is sufficient to detect a significant association between dietary pattern scores and Fe inadequacy.

**Fig. 1:**
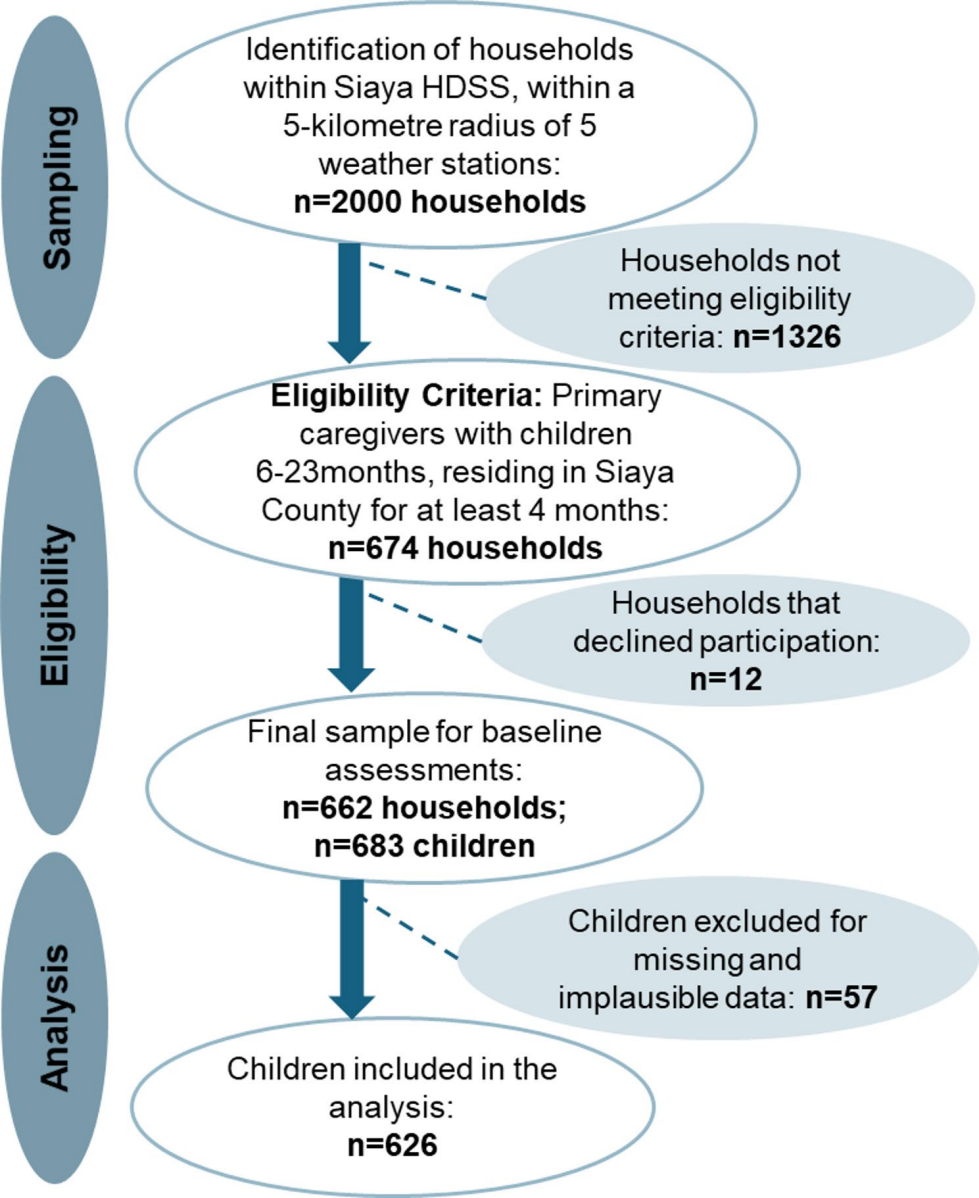
Flow diagram of the recruitment for baseline data collection; HDSS: Health and Demographic Surveillance System

The participating households of the ALIMUS trial were those residing in 385 villages of the Siaya Health and Demographic Surveillance System (HDSS) area. From this pool, a list of households with children below five years of age, residing within a 5-kilometre radius of five weather stations was generated, which yielded a sampling frame of 2,000 households. Households were selected using a probability proportionate to population size (PPS). For the HDSS, a household is defined as an independent socio-economic unit [21]. Figure 1 describes the recruitment process. The eligibility criteria included: having at least one child at the age of complementary feeding (6 months to 23 months); being registered as residents within the HDSS for at least four calendar months before data collection; and having access to water and land to support the intended home gardening component of the ALIMUS intervention. Of the 2,000 households, 674 met the inclusion criteria. 12 households declined participation, resulting in a final sample of 662 households and 683 children examined at baseline (Fig. 1).

### Data collection

#### Dietary assessment

The African-specific Food Propensity Questionnaire (AFPQ) was employed to assess the habitual food intake of the children over the past 6 months. This period spanned from February to June 2021, a period overlapping with the rainy season. The AFPQ is an adapted version of a culture-specific, semi-quantitative tool [25] based on the European Food Propensity Questionnaire, developed in the European Prospective Investigation into Cancer and Nutrition (EPIC) cohort in 2006. It estimates the usual intake of the population [26]. The questionnaire was administered to caregivers, primarily to mothers, by well-trained research assistants with extensive experience in health and nutrition research. The data were collected on tablets using the survey solution platform (HDSS 2.0 susol).

We assessed the frequency of food intake for 134 food items commonly consumed by young children in Kenya. The food items had predefined age-specific portion sizes, estimated by standard household measures from Kenya. The questionnaire inquired whether and how often the child had consumed the listed food items over the past 6 months. We translated the data of each food item into daily intake frequencies and converted those into daily amounts (g/d) [25]. The 134 food items were collated into 29 food groups, based on similarities in culinary use and nutrient composition. We calculated energy intake and nutrient intakes using various nutrient databases for SSA: West African Food Composition Table [27], Kenya Food Composition Table [28], and United States Food and Nutrition Database [29].

#### Assessment of demographic and socio-economic covariables

We administered the HDSS demographic and socio-economic questionnaire module, containing demographic variables (child’s age and sex, mother’s age, place of living, marital status) and socio-economic variables (mother’s education, mother’s occupation, ethnicity, religion, household size, and number of under-fives in the household).

### Data Analysis

#### Missing data handling and descriptive statistics

We analyzed our data using SAS software (version 9.4). We excluded 57 children (8.3%) due to missing, incomplete, or implausible data (energy intake < 100 or > 1900 kcal per day) [30] (Fig. 1). It is not recommended to use multiple imputation for the main outcome variables (here: diet) [31]. Therefore, we kept to the final analytical sample size of 626 participants. To assess the distribution of continuous data, we generated Q-Q plots and performed Shapiro-Wilk tests to calculate z-scores. We also checked for kurtosis and skewness [32]. We present normally distributed data using means ± standard deviations (SDs); otherwise, medians and interquartile ranges (IQR) are shown for non-normally distributed data. Categorical variables were presented as proportions (%) and counts.

## Exploratory dietary patterns

To determine the children’s dietary patterns, we applied Principal Component Analysis using the intake data of 29 food groups (g/d). We employed the PROC FACTOR procedure in SAS 9.4 and an orthogonal rotation to ensure that the pattern scores remained uncorrelated, corresponding to the VARIMAX procedure. In order to select the number of dietary patterns, we assessed the eigenvalues > 1.0, the scree plot (elbow method), and the interpretability of pattern scores, i.e., each pattern score shows at least 4 items with factor loadings ≥ |0.40| [33, 34] (Supplementary Fig. 1). Each child was assigned a score for each identified dietary pattern to reflect their pattern adherence. Each score was calculated by summing the standardized food intake multiplied by the factor loading of this food group. Furthermore, we computed quintiles of each dietary pattern score and calculated the distributions of demographic, socio-economic, and food intake data across these quintiles. Trends across quintiles were determined using linear trend tests [35] and chi-square (*χ*^*2*^*)* tests.

## Adequacy of climate-sensitive micronutrients

To assess adequate intakes of climate-sensitive micronutrients, we calculated the Nutrient Adequacy Ratios (NARs) for zinc, iron, selenium, and vitamin A using the World Health Organization (WHO) cut-offs for these nutrients [36]. NAR is expressed as the individual’s age- and sex-specific intake of the respective nutrient in percentages (capped at 100%) of the corresponding Recommended Daily Allowance (RDA). In addition, we calculated the Mean Adequacy Ratio (MAR) for the four climate-sensitive micronutrients as MAR = sum of NARs/number of nutrients [37]. For each micronutrient and for all four micronutrients together, we calculated the proportion of children with inadequate intakes (defined as NAR or MAR < 80%).

### Associations between dietary patterns and climate-sensitive micronutrients

We used the SAS proc multicollinearity procedure to determine the presence or absence of strong correlations between variables of interest. A variance inflation factor > 10 denotes multicollinearity between the variables. The associations between the dietary pattern scores and the inadequacies of climate-sensitive micronutrients (NAR < 80%, MAR < 80%) were analyzed using logistic regression models. We calculated odds ratios (ORs), their 95% confidence intervals (CIs), and p-values. We fitted three regression models: Model 1 was adjusted for age and sex. Model 2 was further adjusted for maternal age, religion, educational level, marital status, ethnicity, household size, and the number of under-fives. Model 3 was additionally adjusted for energy intake and fiber intake. We performed correction for multiple testing using the Bonferroni-Holm method [38].

### Sensitivity analysis

To check the robustness of our findings [39], we used alternative cut-offs for estimating NARs. In addition to the WHO references, we applied the recommended nutrient intake (RNI) cut-offs of the National Academic Press (NAP) [40]. We compared the proportions of micronutrient inadequacies among children according to the WHO cut-offs and NAP cut-offs. Also, as a subgroup analysis, we excluded non-breastfed children (*n* = 163) and generated the dietary patterns for children who were still breastfed at the time of data collection (*n* = 463). We also repeated the association analyses in this group.

## Results

### Characteristics of the study population

The socio-demographic characteristics and intakes of micronutrients of the total study population (*N* = 626) are presented in Table 1. More than half (54.2%) of the children were boys. The median age of the children was 15 months (IQR: 11–19 months), and the median maternal age was 29 years (IQR: 24–34 years). Two-thirds of the mothers (66.1%) had attained low levels education (primary or no formal school education). Half of the mothers (54.5%) worked as farmers; a quarter were housewives or skilled labourers. The median size of the participating households was 5 persons (IQR: 4–7 persons), and most households had two under-fives (IQR: 1–2). The characteristics were similar between boys and girls, apart from the number of under-fives in the household.

**Table 1:** Characteristics of study participants and their intakes of micronutrients (*N* = 626)

Characteristics	Total	Boys	Girls	
N	626 (100)	339 (54.2)	287 (45.8)	
Child’s age (months)	15 (11–19)	15 (11–19)	15 (10–19)	
Mother’s age (years)	29 (24–34)	29 (24–34)	29 (25–34)	
Mother’s educational status: Elementary	66.1 (414)	65.2 (221)	67.3 (193)	
Mother’s occupation: Farmer	54.2 (330)	54.0 (183)	54.4 (156)	
Mother’s marital status: Married	77.3 (484)	75.2 (255)	79.8 (229)	
Mother’s religion: Christian	42.0 (263)	40.4 (137)	43.9 (126)	
Mother’s ethnicity: Luo	93.6 (586)	92.9 (315)	94.4 (271)	
Number of people in the household	5 (4–7)	5 (4–7)	5 (4–7)	
Number of under-fives in the household	2 (1–2)	1 (1–2)	2 (1–2)	
**Micronutrient intakes**				
Zinc (mg/d)	5.9 (4.1–7.5)	5.9 (4.19–7.5)	6.0 (4.0–7.4)	
Iron (mg/d)	7.3 (5.3–8.7)	7.4 (5.39–8.7)	7.0 (5.0–8.7)	
Selenium (µg/d)	49 (31.8–66.6)	48.1 (31.4–65.3)	50.1 (32.5–68.5)	
Retinol Equivalents (µg/d)	432.4 (279.3–596.2.3.2)	448.1 (285.2–598.0)	424.4 (273.5–585.1)	
**Nutrient adequacy ratios (NARs)**				
Zinc (NAR < 80%)	393 (62.8)	212 (62.5)	181 (63.1)	
Iron (NAR < 80%)	532 (85.0)	290 (85.5)	242 (84.3)	
Selenium (NAR < 80%)	68 (10.9)	41 (12.1)	27 (9.4)	
Retinol Equivalent (NAR < 80%)	195 (31.2)	100 (29.5)	95 (33.1)	
Mean Adequacy Ratio < 80%	312 (49.8)	162 (47.8)	150 (52.3)	

Nutrient adequacy ratios (NARs) were calculated using the WHO-recommended nutrient intake (RNI) cut-offs. Data are presented as medians (with interquartile ranges, IQR) for continuous variables and as counts (proportions, %) for categorical variables.

Regarding micronutrient intake, there were no differences between boys and girls. Inadequate intakes of iron were seen in 85%, followed by zinc (63%), vitamin A (31%), and selenium (11%). Half of the children (50%) did not meet the adequacy threshold for the four micronutrients (zinc, iron, selenium, and vitamin A; MAR < 80%) (Table 1). As an alternative, we defined the micronutrient adequacy using the NAP cut-offs for intake. This approach classified 25% of the children as not achieving the adequacy threshold for the four micronutrients (MAR < 80%) (Supplementary Table 2). According to the NAP cut-offs, the proportions of children classified as having inadequate intakes were 42% for iron, 6% for zinc, 27% for vitamin A, and 25% for selenium.

### Food intake and dietary patterns

**Fig. 2:**
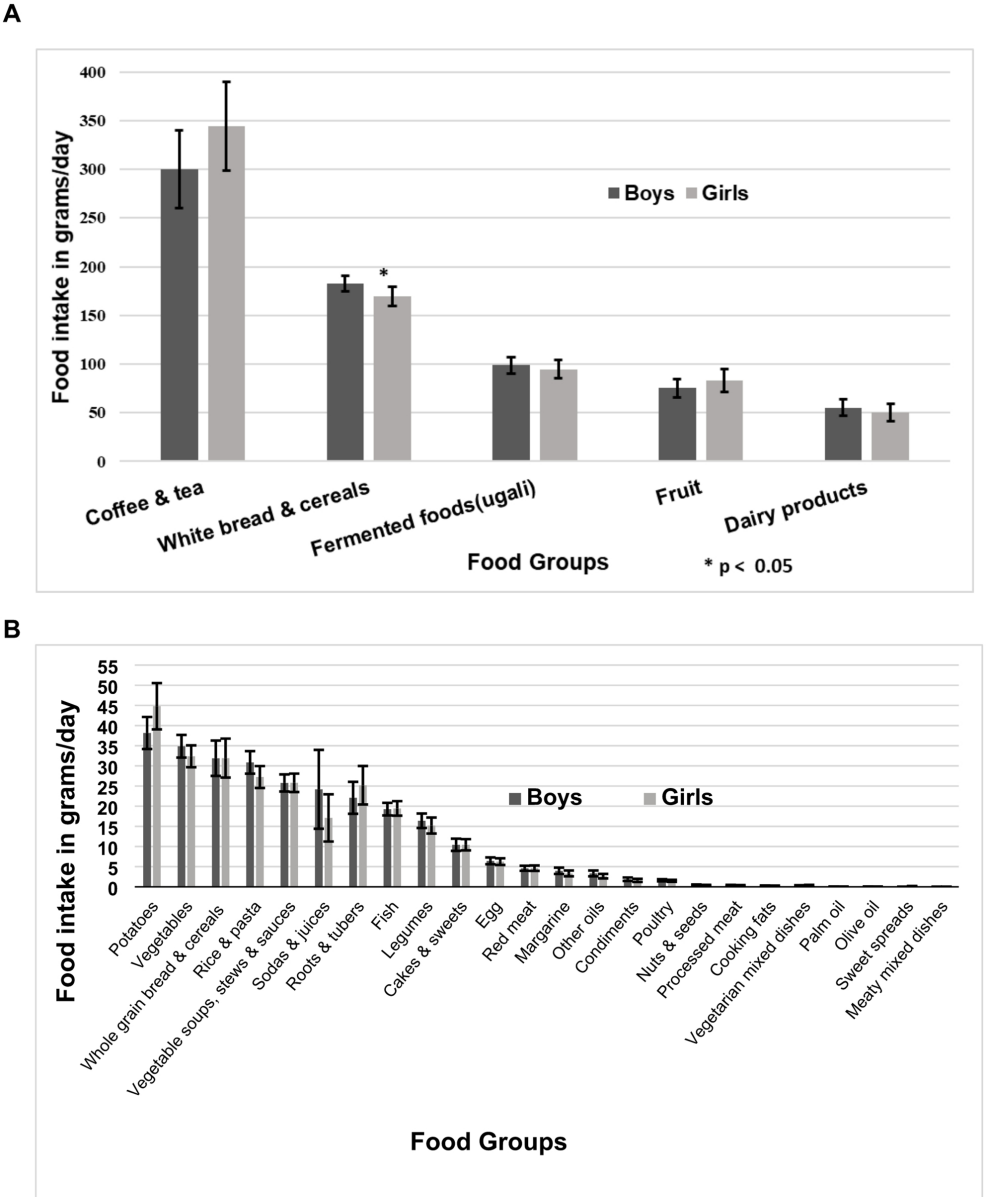
Means and standard deviations (error bars) for the intakes of 29 food groups among children aged 6 to 23 months in Siaya county, (**A**) daily consumption of ≥ 50 g/d and (**B**) daily consumption of < 50 g/d

Of the 29 food groups, tea and coffee (as a ready-to-drink formulation) were most frequently consumed, with a mean of 320 ± 383 g/day. Other commonly consumed food groups included white breads and cereals (mean 177 ± 80 g/d), fermented maize (ugali) (mean 97 ± 78 g/d), fruits (mean 79 ± 94 g/d), and dairy products (mean 53 ± 77 g/d) (Fig. 2). The least consumed food groups were processed meats, cooking fats, vegetarian mixed dishes, palm oil, olive oil, sweet spreads, and meaty mixed dishes. Generally, boys consumed more amounts across the food groups than girls, except for tea, coffee, fruits, potatoes, and roots and tubers. The differences in food intake between boys and girls were, however, not statistically significant with the exception of white breads and cereals (*p* = 0.04).

**Fig. 3:**
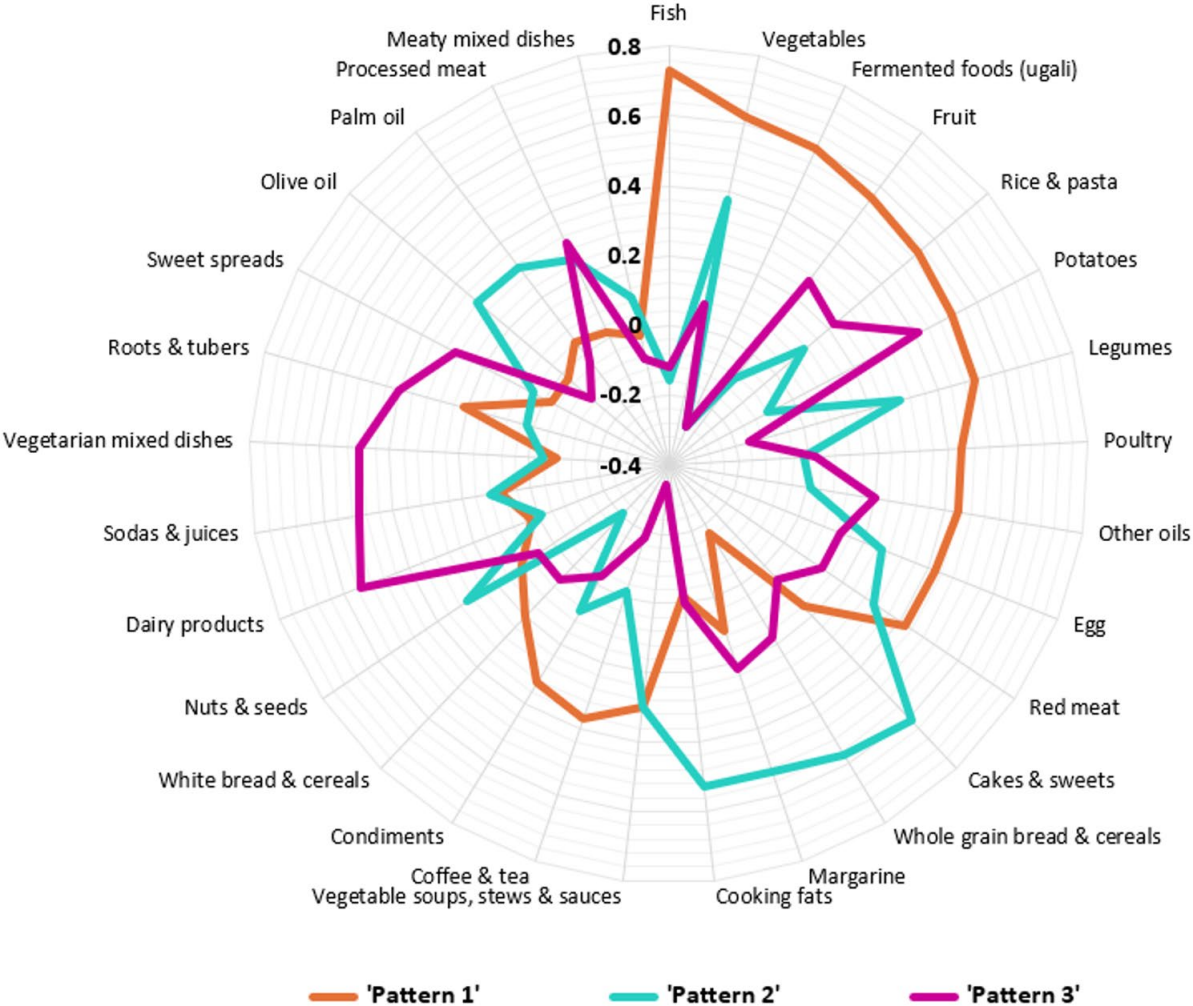
Rotated factor loadings of three dietary patterns among 626 children in Siaya county. Food groups with factor loadings ≥ |0.40| were considered major contributors to the pattern score.

We identified three distinct dietary patterns and displayed them in a spider web diagram (Fig. 3). The patterns were labelled ‘Pattern 1’, ‘Pattern 2’, and ‘Pattern 3’, and together accounted for 27% of the variation in food intake.

Pattern 1 accounted for 12.6% of the total variation in food intake, and was characterized by high intakes of fish, vegetables, fermented foods (ugali from millet, cassava and fermented maize flours), fruit, rice, pasta, potatoes, legumes, poultry, red meat, and other oils (Supplementary Table 3). Pattern 2 explained 8.0% of the variation and had high positive loadings of cakes and sweets, whole-grain breads and cereals, margarine, and cooking fat (Supplementary Table 3). Pattern 3 accounted for 6.4% of the variation in food intake and was characterized by strong adherence to milk and dairy products, sodas and juices, vegetarian mixed dishes, and roots and tubers (Supplementary Table 3).

Participants in the highest quintile of ‘pattern 1’ had higher energy intake, were older, more frequently belonged to households with educated mothers (post-elementary education), and had fewer household members than those in lower quintiles (Supplementary Table 4). In higher quintiles of ‘pattern 2’, children had higher energy intakes and were older than children in lower quintiles (Supplementary Table 5). Lastly, for ‘pattern 3’, children in higher quintiles were older, belonged to the Luo ethnic group, and consumed more energy than those in the lower quintiles (Supplementary Table 6). Overall, higher adherence to all three dietary patterns was associated with higher energy intake and older age. For the subgroup analysis among children who were still breastfed (*n* = 463), three patterns were derived, which showed similar features to those in the total study population (Supplementary Table 7). Also, the total explained variation in food intake was similar in the subgroup compared to the full sample (29% vs. 27%).

### Associations of dietary patterns with climate-sensitive micronutrients

The associations between ‘pattern 1’ and the adequacy of climate-sensitive micronutrients are shown in Table 2. Adherence to this pattern was inversely associated with zinc inadequacy (OR: 0.57; 95% CI: 0.38, 0.86), iron inadequacy (OR: 0.20; 95% CI: 0.11, 0.37), vitamin A inadequacy (OR: 0.40; 95% CI: 0.25, 0.65), and overall micronutrient inadequacy (MAR < 80%) (OR: 0.20; 95% CI: 0.11, 0.37), but not with selenium inadequacy (OR: 0.56; CI: 0.28, 1.15).

Furthermore, for the associations between ‘pattern 2’ and climate-sensitive micronutrients (Table 2), we did not find associations with zinc and selenium inadequacies. Yet, this pattern increased the odds of iron inadequacy (OR: 1.54; 95% CI: 1.10, 2.16) and was inversely associated with vitamin A inadequacy (OR: 0.39; 95% CI: 0.26, 0.58). For the associations between ‘pattern 3’ and inadequacy of climate-sensitive micronutrients, there were direct associations with zinc inadequacy (OR: 1.87; 95% CI: 1.43, 2.44), selenium inadequacy (OR: 1.95; 95% CI: 1.25, 3.06), and overall micronutrient inadequacy (OR: 2.41; 95% CI: 1.73, 3.37). There was no association with iron inadequacy and an inverse association with vitamin A inadequacy (OR: 0.55; 0.36, 0.85) (Table 2). Most associations remained statistically significant after correction for multiple testing using the Bonferroni-Holm method. The associations between 'pattern 1' and zinc inadequacy and between 'pattern 2' and iron inadequacy showed borderline significance after correction for multiple testing.

In the subgroup analysis, similar trends were observed for the three dietary patterns in relation to micronutrient inadequacies among children still being breastfed (Supplementary Table 8). ‘Pattern 1’ was inversely associated with inadequacy of all climate-sensitive micronutrients, including selenium. Unlike in the total sample, ‘pattern 2’ was not significantly associated with iron inadequacy (*p* = 0.07). Lastly, the association between ‘pattern 3’ and inadequacies of zinc, vitamin A, selenium, and overall micronutrients were still discernible in this subgroup.

**Table 2:** Associations of three dietary patterns with the inadequacies of climate-sensitive micronutrients among 626 children aged 6–23 months

Outcome	*n* (%)	*Crude*		*Model 1*		*Model 2*		*Model 3*	
		OR (95% CI)	*p*-value	OR (95% CI)	*p*-value	OR (95% CI)	*p*-value	OR (95% CI)	*p*-value
‘Pattern 1’									
Zn inadequacy	393 (62.8)	0.14 (0.11, 0.20)	< 0.0001	0.15 (0.11, 0.21)	< 0.0001	0.16 (0.11, 0.22)	< 0.0001	0.57 (0.38, 0.86)	0.008
Fe inadequacy	532 (85.0)	0.10 (0.06, 0.15)	< 0.0001	0.10 (0.06, 0.17)	< 0.0001	0.10 (0.06, 0.16)	< 0.0001	0.20 (0.11, 0.37)	< 0.0001
Se inadequacy	68 (10.9)	0.20 (0.13, 0.29)	< 0.0001	0.15 (0.09, 0.24)	< 0.0001	0.12 (0.07, 0.21)	< 0.0001	0.56 (0.28, 1.15)	0.114
Vit A inadequacy	195 (31.2)	0.14 (0.10, 0.19)	< 0.0001	0.15 (0.11, 0.21)	< 0.0001	0.16 (0.11, 0.22)	< 0.0001	0.40 (0.25, 0.65)	0.0002
MAR < 80%	312 (49.8)	0.11 (0.08, 0.16)	< 0.0001	0.12 (0.08, 0.17)	< 0.0001	0.12 (0.08, 0.17)	< 0.0001	0.20 (0.11, 0.37)	< 0.0001
**‘Pattern 2’**									
Zn inadequacy	393 (62.8)	0.78 (0.67, 0.92)	0.003	0.90 (0.76, 1.07)	0.228	0.88 (0.73, 1.06)	0.167	0.83 (0.64, 1.08)	0.165
Fe inadequacy	532 (85.0)	1.00 (0.80, 1.24)	0.991	1.20 (0.95, 1.53)	0.128	1.16 (0.91, 1.47)	0.234	1.54 (1.10, 2.16)	0.011
Se inadequacy	68 (10.9)	0.83 (0.63, 1.10)	0.199	0.89 (0.66, 1.19)	0.421	0.93 (0.68, 1.26)	0.627	0.87 (0.53, 1.45)	0.603
Vit A inadequacy	195 (31.2)	0.52 (0.41, 0.65)	< 0.0001	0.62 (0.49, 0.79)	< 0.0001	0.60 (0.47, 0.77)	< 0.0001	0.39 (0.26, 0.58)	< 0.0001
MAR < 80%	312 (49.8)	0.69 (0.58, 0.82)	< 0.0001	0.84 (0.70, 1.01)	0.060	0.82 (0.68, 0.99)	0.042	0.94 (0.65, 1.36)	0.742
**‘Pattern 3’**									
Zn inadequacy	393 (62.8)	0.85 (0.72, 1.00)	0.043	0.85 (0.72, 1.00)	0.052	0.86 (0.72, 1.03)	0.094	1.87 (1.43, 2.44)	< 0.0001
Fe inadequacy	532 (85.0)	0.62 (0.51, 0.77)	< 0.0001	0.63 (0.51, 0.78)	< 0.0001	0.63 (0.50, 0.80)	< 0.0001	1.08 (0.82, 1.41)	0.590
Se inadequacy	68 (10.9)	0.86 (0.64, 1.15)	0.304	0.86 (0.63, 1.16)	0.308	0.92 (0.67, 1.26)	0.592	1.95 (1.25, 3.06)	0.004
Vit A inadequacy	195 (31.2)	0.53 (0.41, 0.67)	< 0.0001	0.46 (0.35, 0.61)	< 0.0001	0.46 (0.35, 0.61)	< 0.0001	0.55 (0.36, 0.85)	0.006
MAR < 80%	312 (49.8)	0.78 (0.66, 0.93)	0.006	0.76 (0.63, 0.92)	0.005	0.77 (0.63, 0.94)	0.010	2.41 (1.73, 3.37)	< 0.0001

Logistic regression was used to calculate odds ratios (ORs), 95% confidence intervals (CIs), and p-values.

Zn = zinc, Fe = iron, Se = selenium, Vit A = vitamin A, MAR = Mean Adequacy Ratio.

Crude: no adjustments;

Model 1: adjusted for child’s sex and age;

Model 2: Model 1 + maternal age, maternal education, maternal occupation, maternal marital status, number of under-fives, household size;

Model 3: Model 2 + energy intake (kcal/d), dietary fibre (g/d).

## Discussion

In this cross-sectional study, we identified food group consumption, dietary patterns, the adequacy of micronutrient intakes, as well as the associations between dietary patterns and climate-sensitive micronutrient inadequacies among children 6–23 months. The dominating food groups were coffee and tea, white bread and cereals, fermented maize (ugali), and dairy. The proportions of children with inadequate intakes of micronutrients were 85% for iron, 63% for zinc, 31% for vitamin A, 11% for selenium, and 50% for all four micronutrients. In the multiple-adjusted models, ‘pattern 1’ was associated with lower odds of inadequate intakes of climate-sensitive micronutrients, except for selenium. 'Pattern 2' was associated with higher odds of inadequate intake of iron and lower odds of inadequate intake of vitamin A. Lastly, ‘pattern 3’ showed increased odds of inadequate intakes of zinc and selenium and all four climate-sensitive micronutrients.

### Food consumption and dietary patterns

Comparing our findings with those of a previous study among 6–59-months-old children in the Kwale rural community in Kenya, using cluster analysis [41], we observed similarities in dietary patterns, namely ‘protein-rich’, ‘traditional’, and ‘traditional complemented by breastfeeding’. The ‘protein-rich’ cluster is characterized by similar food groups as ‘pattern 1’ in the present study. The ‘traditional diet’ in Kwale matches the Siaya ‘pattern 3’, because the two have fewer protein-rich foods. In addition, the identified dietary patterns confirm that fish and vegetables alongside starchy foods form the basis of the children’s diets [20]. In a formative study of the ALIMUS project in rural Burkina Faso, Mank et al. have used PCA and extracted four dietary patterns among young children (6–59 months), namely, ‘leaves-based diet’, ‘beans and poultry-based diet’, ‘maize and fish-based diet’, and ‘millet and meat-based diet’ [42]. The four dietary patterns contrast those extracted in Siaya County in the characteristic food groups, except for the ‘maize and fish-based’ pattern, which resembles the emphasis on maize and fish in ‘pattern 1’. Some food groups with high factor loadings (okra) in the ‘maize and fish-based diet’ for the Burkina Faso population, however, were rarely consumed by children in Kenya, reflecting the complexity of comparing different dietary patterns across different cultures and regions [35, 43]. The explained variation in food intake observed in our study was similar to that seen in other nutritional epidemiological analyses that used the same exploratory approach [25, 42].

For the analysis of consumption in relation to socioeconomic status (SES) proxies, high mothers’ education and low household size were associated with high adherence to ‘pattern 1’ but not 'pattern 2' and 'pattern 3'. 'Pattern 1' is characterized by intakes of various food groups containing starchy, protein and vegetable groups. Though specific diet quality indices (diversity and variety scores) were not assessed in this study, ‘pattern 1’ can be viewed as having more variety compared to the other two patterns. Caregivers’ education and literacy contribute to food choices and positively correlate with increased odds of appropriate child feeding practices across SSA [44, 45], and increased consumption of fruits and vegetables and energy-dense foods in other regions [46]. Therefore, it is not surprising that educated mothers in our study prefer ‘pattern 1’ for its perceived health benefit. In other studies, however, high SES was associated with increased odds of consumption of unhealthy foods (sweet beverages, zero-fruit and vegetable consumption) [47], while belonging to a rural area was attributed to low odds of consumption of unhealthy foods among children. This contradicts our findings as neither ‘pattern 2’ nor ‘pattern 3’, characterized by consumption of cakes and sweets, high fats and sweet beverages, showed an associations with SES proxies. Thus, other factors like media exposure, assessed in other studies [48], likely contributed to the observed variation.

### Dietary patterns in relation to adequate intakes of climate-sensitive micronutrients

The occurrence of inadequate intake of iron (85%) in our study region was considerably higher than the national average among under-fives (22%) [4]. Using linear programming analysis, Ferguson et al. have described iron and zinc as problematic nutrients in Kenya. The authors argue that the children’s adequacy (100% RNI) is difficult to attain using nutritionally best possible modelled diets from the locally available complementary foods in Vihiga and Kitui counties, Kenya [39]. Corroborating Ferguson’s results, iron and zinc were identified as limiting micronutrients in children’s diets in the same county (Vihiga), with an average probability of adequacy below 30% [49]. In other rural settings of the Global South (Burundi, India, South Africa), young children (6–59 months), who consume less than four food groups per day, are found to have increased odds of iron deficiency [50, 51], while those with high scores for adhering to complementary feeding indicators have lower odds of anemia [52]. Other common reasons for the inadequacy of climate-sensitive micronutrients include frequent infectious diseases, unstable food access and availability, and disturbed intestinal absorption [1, 47, 53]. Still, authors of a qualitative study in Siaya have argued that cultural factors and ethnicity contribute to the low intakes of animal-source proteins, such as eggs, fish, and other meats, which may have an impact on the adequacy of protein in general, as well as iron and zinc, in children [54]. These interrelated environmental and socio-cultural factors underscore the importance of integrated nutrition-hygiene-health interventions in improving child micronutrient status. Addressing these determinants through coordinated public health actions could enhance the impact of nutrition-specific programs and strengthen advocacy for child health and development [1].

With regard to the associations between dietary patterns and the adequacy of climate-sensitive micronutrients, the strengths of these associations were in ranges that may buffer the projected micronutrient losses from climate change (4–15%) [11, 12]. Our estimates were highly significant, although their accuracy was varying. Acknowledging that we performed analysis on secondary outcomes, our findings may still be relevant for public health nutrition. We found that ‘pattern 1’ was associated with lower odds of iron inadequacy. In comparison, we found increased odds of iron inadequacy by 54% in children, who strongly adhered to ‘pattern 2’. Iron is an essential mineral that supports oxygen transport to the body cells and plays a major role in brain development. Iron deficiency can lead to anaemia, characterised by fatigue, reduced performance, and impaired neurocognitive development. Iron deficiency anaemia is considered a global health concern, it poses health risks across the life cycle [55]. 'Pattern 1' was mainly characterized by animal-based foods, fermented foods, green vegetables, and fruits, including citrus fruits, and legumes. Most of these foods are rich in heme and non-heme iron and have iron enhancers, such as citrus fruits [40], which could explain their protective association with intakes of iron. On the other hand, ‘pattern 2’ had high factor loadings for whole-grain products. Such foods contain high concentrations of phytates and polyphenols, which inhibit intestinal iron absorption and bioavailability [4]. This could explain why this pattern was associated with an increased odds of inadequate iron intake.

For zinc, around two-thirds of children in our study had inadequate intakes; this was lower than the most recent biomarker-based estimates from Kenya (82%) [4, 56]. Zinc is a mineral involved in cellular activities (DNA and RNA synthesis) and mediates human hormonal actions [57]. Zinc deficiency is associated with impaired fertility, stunted growth, weakened resistance to infections, and early death [58]. Yet, clinically overt zinc-deficiency syndromes (e.g., acrodermatitis enteropathica) are rare and mostly congenital [56]. Children following ‘pattern 1’ had reduced odds of inadequate zinc intake by 43%. This beneficial relationship might be explained by the high factor loadings of green vegetables and red meat, containing considerable amounts of zinc. Also, this pattern had low factor loadings for whole-grain products that contain appreciable quantities of zinc-chelating compounds, and hence, zinc absorption may be supported [59]. In contrast, one SD increase in ‘pattern 3’ score increased the odds of inadequate zinc intake by twofold. In fact, this pattern was characterized by a high intake of milk and sugary foods, which are low in zinc [59]. Previous studies support this finding, indicating that children consuming sugar-sweetened beverages have low serum zinc levels (< 10.7 µmol/L), while those consuming more than 15 g/day of meat have high zinc concentrations [60].

Regarding selenium, the proportion of children with inadequate intake was 10% in our study population. We did not find any data on national selenium levels among children in Kenya. One study with 183 children from central Kenya highlands has reported that 87% were at risk of selenium deficiency, defined by low selenium hair concentration [61]. Similar numbers (96%) have been reported across SSA in studies involving selenium serum biomarkers for child populations [62]. Selenium is a mineral involved in the modulation of multiple cell functions, and selenium deficiencies are associated with cognitive impairments, poor immunity, and increased morbidity [63, 64].

In the full sample of our study, ‘pattern 1’ and ‘pattern 2’ did not seem to affect adequate intakes of selenium. Yet, in the breastfed sample, the former pattern was associated with 72% reduced odds of inadequate selenium intake. Further, ‘pattern 3’, characterized by low factor loadings of animal-source protein, was associated with an increased odds of inadequate selenium intake. In fact, animal-source foods and breastmilk are rich in selenium, which could partially explain our observations [65]. At the same time, soil and landscape features determine the selenium concentration in crops used for complementary foods, and our observations possibly reflect the dilution of selenium in such crops as a climate-sensitive micronutrient [66]. Variations in dietary selenium supply and intake, and linkages to their geospatial locations, have been documented in SSA [61, 63, 66]. Nonetheless, the low factor loading for animal foods and grain products in ‘pattern 3’ could explain the increased risk of inadequate selenium intake.

For vitamin A, 31% of the children in this study population showed inadequate intake. This compares to the national average of 9% in the same age group [4], but might be overestimated due to the lack of data on vitamin A content in the breastmilk of this study population [67]. Our study revealed significant inverse associations of all three dietary patterns with the odds of inadequate intakes of vitamin A. Deficiencies of this micronutrient are associated with increased risks of infection, reduced vision, and poor growth in children [4, 68]. All three patterns showed high factor loadings for foods with retinol from animal sources (egg yolk, fish) or with beta-carotene from plant sources (yellow-fleshed roots, fruits, and dark green vegetables). Processed foods also provide an additional source of vitamin A from fortified flours and margarine [4]. Hence, the multiple vitamin A sources from the different patterns were likely to contribute to adequate vitamin A intake, thereby driving the protective associations. Our findings align with a previous study in India, which observed that Indian children (6–59 months) consuming foods lacking vitamin A rich sources (retinol intake < 400 µg/d and beta-carotene intake < 3200 µg/d), have more than twice the odds of vitamin A deficiency (serum retinol < 0.7 µmol/L) compared to those achieving the recommended dietary allowances (RDAs) for vitamin A [69].

### Strengths and limitations

Our findings need to be interpreted on the background of the study’s strengths and limitations. Despite the fact that we excluded a few children from the analysis, the sampling procedure provided a representative dataset for children aged 6–23 months living in the Siaya HDSS, and findings may thus be generalizable to the population of under-fives in this county. Cross-sectional studies are prone to reverse causation and recall bias. Yet, in the present study, there was no reason to believe that micronutrient inadequacies on a subclinical level have influenced the feeding behaviour and the responses of caregivers during interviews. A major limitation was the estimation of micronutrient intakes (outcomes) from the same assessment tool that was used to measure food intakes for dietary pattern construction (exposures). This may have introduced multicollinearity. The AFPQ was tailored to the culinary practices and cultural context of the study participants, including portion sizes and recipes. However, we used this tool for the first time among young children in Kenya, which might have created over- as well as underestimation. This semi-quantitative assessment tool allowed us to calculate energy and nutrient intakes from food consumption. Admittedly, the AFPQ is a retrospective tool that may underestimate the intakes of seasonal foods and absolute food intake due to the finite list of food groups. Still, all participants may carry the same measurement error, which allowed us to rank the study participants according to their intakes. In addition, the micronutrient supply from other sources, such as mineral water and breastmilk, cannot be estimated by the AFPQ. Finally, we accounted for demographic and socio-economic confounders that are known to be relevant for dietary behaviour in the Global South context. Yet, we cannot exclude residual and unmeasured confounding.

### Public health relevance

Our cross-sectional analysis makes a unique contribution to the literature on the associations between dietary patterns and climate-sensitive micronutrients in young children living in rural sub-Saharan Africa. We demonstrate that certain dietary patterns may be more beneficial for meeting the required supply of climate-sensitive micronutrients than others. Clearly, these findings need to be verified in studies that use independent assessment tools to relate dietary intake with biomarkers of nutrient status across different seasons. Yet, the observed associations between dietary patterns and climate-sensitive micronutrient adequacy can inform the development of nutrition-specific interventions for under-fives in Siaya County in the era of climate crisis [39, 42]. This may include reviewing feeding guidelines and strengthening access to these guidelines for the health workforce and caregivers, focusing on key messages related to complementary feeding. Beyond this access, it is also vital to actively enhance nutrition literacy and fluency to support the adequate intake of climate-sensitive micronutrients among under-fives [70]. These approaches need to be integrated into a comprehensive policy framework for sustainable food systems, spanning agricultural production, food processing, storage, and transport, as well as food provision and consumption [10, 71]. These strategies will contribute to creating resilience towards the impacts of climate change on child nutrition in this area.

## Conclusion

Using exploratory methods, three dietary patterns were identified among children at the age of complementary feeding in Siaya County, Western Kenya. This study found frequent occurrences of inadequate intakes of iron, zinc, and vitamin A among young children in Siaya County. 'Pattern 1' is the most promising diet for reducing inadequate intakes of all climate-sensitive micronutrients. A change towards more modernized diets with processed and starchy foods may contribute to the deterioration of inadequate intakes. Combined with the anticipated negative impacts of climate change on the quality of food crops, the nutritional status of children in Siaya county may worsen over the next years. Future studies should explore behavioural change strategies as part of nutrition-sensitive and nutrition-specific interventions that target caregivers’ nutrition literacy and fluency to improve their knowledge, skills, and complementary feeding practices.

## Supplementary Information

Supplementary Material 1.

Supplementary Material 2.

## Data Availability

The data that support this study’s findings are available from the Steering Committee of the DFG-funded Research Unit “Climate change and health in sub-Saharan Africa,” coordinated by Ina Danquah: ina.danquah@uni-bonn.de. However, restrictions apply to the availability of these data, which were used under license for the current study and so are not publicly available. Data are, however, available from the authors upon reasonable request and with permission of the Steering Committee of the DFG-funded Research Unit.

## Abbreviations

AFPQ: African Food Propensity Questionnaire
HDSS: Health and Demographic Surveillance System
NAR: Nutrient Adequacy Ratio
MAR: Mean Adequacy Ratio
PCA: Principal Component Analysis
WHO: World Health Organization
STROBE: Strengthening the Reporting of Observational Studies in Epidemiology
