# Supplementary material for: Associations between dietary patterns and adequate intake of climate-sensitive micronutrients among young children living in Siaya County, Kenya: Findings from the ALIMUS study baseline data
Source: J Health Popul Nutr. 2026 Mar 26;45:110. doi: 10.1186/s41043-026-01293-y (PMC13047798; doi:10.1186/s41043-026-01293-y)
Supplement: Supplementary file 2 — Supplementary Material 2. [file 41043_2026_1293_MOESM2_ESM.docx]

**Supplementary Figure 1: Scree plot for the extraction of three dietary patterns derived among 626 children aged 6-23 months living in Siaya County, Kenya**


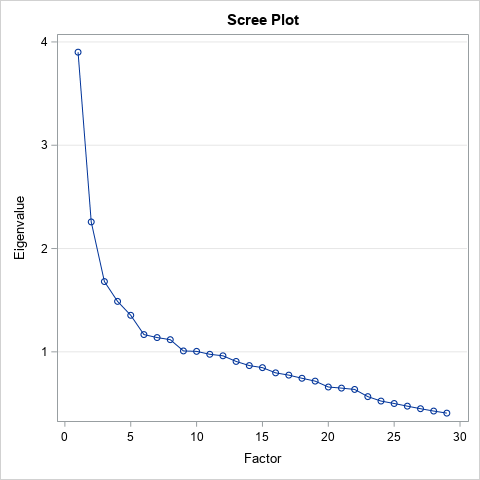


**Supplementary Table 2: Climate-sensitive micronutrient adequacies among 626 children in Siaya County**

| **Nutrients** | **Total (N=626)** | | **Males (n=339)** | | **Females (n=287)** | |
| --- | --- | --- | --- | --- | --- | --- |
| Zinc (NAR < 80%) | 38 | 6.1 | 20 | 5.9 | 18 | 6.3 |
| Iron (NAR < 80%) | 260 | 41.5 | 132 | 38.9 | 128 | 44.6 |
| Selenium (NAR < 80%) | 158 | 25.2 | 88 | 26.0 | 70 | 24.4 |
| Retinol Equivalent (NAR < 80%) | 170 | 27.2 | 85 | 25.1 | 85 | 29.6 |
| MAR < 80% | 158 | 25.2 | 78 | 23.0 | 80 | 27.9 |

Nutrient Adequacy Ratios (NARs) were calculated based on the cut-offs for recommended nutrient intakes (RNI) from the National Academic Press. Data are presented as counts (proportions, %).

**Supplementary Table 3: Rotated factor loadings of three dietary patterns among children in Siaya County (n = 626)**

| **Food categories** | **Pattern 1** | **Pattern 2** | **Pattern 3** |
| --- | --- | --- | --- |
| Fish | **0.73** | -0.16 | -0.12 |
| Vegetables | **0.62** | 0.38 | 0.07 |
| Fermented foods (ugali) | **0.60** | -0.28 | -0.28 |
| Fruit | **0.56** | -0.09 | 0.26 |
| Rice & pasta | **0.54** | 0.11 | 0.22 |
| Potatoes | **0.52** | -0.08 | 0.41 |
| Legumes | **0.51** | 0.29 | -0.16 |
| Poultry | **0.44** | -0.01 | 0.02 |
| Other oils | **0.44** | 0.01 | 0.20 |
| Egg | **0.42** | 0.26 | 0.13 |
| Red meat | **0.42** | 0.31 | 0.13 |
| Cakes & sweets | 0.16 | **0.61** | 0.05 |
| Whole grain bread & cereals | -0.17 | **0.57** | 0.18 |
| Margarine | 0.10 | **0.53** | 0.22 |
| Cooking fats | -0.02 | **0.53** | 0 |
| Vegetable soups, stews & sauces | 0.30 | 0.30 | -0.34 |
| Coffee & tea | 0.37 | -0.02 | -0.18 |
| Condiments | 0.33 | 0.09 | -0.03 |
| White bread & cereals | 0.20 | -0.21 | 0.05 |
| Nuts & seeds | 0.11 | 0.3 | 0.05 |
| Dairy products | 0.02 | -0.01 | **0.55** |
| Sodas & juices | 0.09 | 0.12 | **0.50** |
| Vegetarian mixed dishes | -0.08 | -0.04 | **0.49** |
| Roots & tubers | 0.21 | 0.02 | **0.40** |
| Sweet spreads | -0.02 | 0.04 | 0.29 |
| Olive oil | -0.02 | 0.32 | -0.11 |
| Palm oil | 0.04 | 0.31 | -0.03 |
| Processed meat | 0.02 | 0.25 | 0.30 |
| Meaty mixed dishes | -0.02 | 0.09 | -0.09 |
| Eigenvalues percentage | 3.7 | 2.3 | 1.9 |
| Eigenvalues percentage contribution (% explained variance) | 12.6 | 8.0 | 6.4 |
| Total explained variance by all patterns | **27.03%** |  |  |

*Food groups with ≥ |0.40| factor loadings were considered major contributors to the pattern scores*

**Supplementary Table 4: Socio-demographic characteristics and food group intakes across quintiles of ‘Pattern 1’**

| **Characteristics** | **Q1 (n=120)** | | **Q2 (n=126)** | | **Q3 (n=126)** | | **Q4 (n=127)** | | **Q5 (n=127)** | | **p for trend** |
| --- | --- | --- | --- | --- | --- | --- | --- | --- | --- | --- | --- |
| Male sex | 62 | 51.67 | 67 | 53.17 | 77 | 61.11 | 66 | 51.97 | 67 | 52.76 | 0.533 |
| Age (months) | 10.5 | (7, 17) | 14 | (10, 19) | 15 | (11, 18) | 16 | (12, 20) | 17 | (14, 21) | <.0001 |
| Mother's age (years) | 29 | (24, 34) | 29 | (26, 34) | 29.1 | (23, 35) | 30 | (24, 34) | 28 | (24, 32) | 0.454 |
| Elementary education | 95 | 79.17 | 90 | 71.43 | 86 | 68.25 | 77 | 60.63 | 66 | 51.97 | <.0001 |
| Occupation: Housewife | 24 | 20.00 | 29 | 23.02 | 22 | 17.46 | 17 | 13.39 | 26 | 20.47 | 0.492 |
| Farmer | 72 | 60.00 | 66 | 52.38 | 64 | 50.79 | 72 | 56.69 | 65 | 51.18 |  |
| Skilled labourer | 17 | 14.17 | 26 | 20.63 | 28 | 22.22 | 29 | 22.83 | 25 | 19.69 |  |
| Business | 7 | 5.83 | 5 | 3.97 | 12 | 9.52 | 9 | 7.09 | 11 | 8.66 |  |
| Marital status: married | 89 | 74.17 | 97 | 76.98 | 100 | 79.37 | 99 | 77.95 | 99 | 77.95 | 0.903 |
| Religion: Protestants | 19 | 15.83 | 17 | 13.49 | 29 | 23.02 | 22 | 17.32 | 24 | 18.90 | 0.013 |
| Catholic | 18 | 15.00 | 35 | 27.78 | 31 | 24.60 | 26 | 20.47 | 42 | 33.07 |  |
| Mixed | 40 | 33.33 | 44 | 34.92 | 44 | 34.92 | 45 | 35.43 | 31 | 24.41 |  |
| No religion  /Traditionalist | 43 | 35.83 | 30 | 23.81 | 22 | 17.46 | 34 | 26.77 | 30 | 23.62 |  |
| Ethnic group: Luo | 110 | 91.67 | 117 | 92.86 | 120 | 95.24 | 119 | 93.70 | 120 | 94.49 | 0.809 |
| Other ethnic groups | 10 | 8.33 | 9 | 7.14 | 6 | 4.76 | 8 | 6.30 | 7 | 5.51 |  |
| Number of under-fives | 2 | (1, 2) | 1.32 | (1, 2) | 2 | (1, 2) | 2 | (1, 2) | 2 | (1, 2) | 0.077 |
| Household size | 6 | (5, 7) | 6 | (4, 7) | 6 | (4, 7) | 5 | (4, 7) | 5 | (4, 6) | 0.009 |
| Energy intake (kcal/d) | 599 | (391, 876) | 773.6 | (555, 1052) | 1024 | (799, 1228) | 1121.7 | (903, 1397) | 1485.4 | (1262, 1644) | <.0001 |

Data presented as medians and interquartile ranges (IQR) for continuous variables and as counts (proportions, %) for categorical variables. P values for categorical variables were calculated by x ^2^ test.

**Supplementary Table 5: Socio-demographic characteristics and food group intakes across quintiles of ‘Pattern 2’**

| **Characteristics** | **Q1 (n=125)** | | **Q2 (n=126)** | | **Q3 (n=127)** | | **Q4 (n=123)** | | **Q5 (n=125)** | | **P for trend** |
| --- | --- | --- | --- | --- | --- | --- | --- | --- | --- | --- | --- |
| Male sex | 61 | 48.80 | 67 | 53.17 | 72 | 56.69 | 70 | 56.91 | 69 | 55.20 | 0.689 |
| Age (months) | 12 | (8, 18) | 13 | (8, 18) | 15 | (11, 19) | 16 | (12, 19) | 17 | (14, 21) | <.0001 |
| Mother's age (years) | 29 | (23, 34) | 29.6 | (25, 35) | 28 | (22, 34) | 30 | (26, 34) | 28 | (25, 32) | 0.087 |
| Elementary education | 82 | 65.60 | 84 | 66.67 | 85 | 66.93 | 76 | 61.79 | 87 | 69.60 | 0.777 |
| Occupation: Housewife | 21 | 16.80 | 26 | 20.63 | 27 | 21.26 | 20 | 16.26 | 24 | 19.20 | 0.804 |
| Farmer | 79 | 63.20 | 66 | 52.38 | 63 | 49.61 | 67 | 54.47 | 64 | 51.20 |  |
| Skilled labourer | 18 | 14.40 | 26 | 20.63 | 26 | 20.47 | 28 | 22.76 | 27 | 21.60 |  |
| Business | 7 | 5.60 | 8 | 6.35 | 11 | 8.66 | 8 | 6.50 | 10 | 8.00 |  |
| Marital status: married | 98 | 78.40 | 101 | 80.16 | 100 | 78.74 | 95 | 77.24 | 90 | 72.00 | 0.584 |
| Religion: Protestants | 24 | 19.20 | 21 | 16.67 | 19 | 14.96 | 22 | 17.89 | 25 | 20.00 | 0.653 |
| Catholic | 33 | 26.40 | 34 | 26.98 | 24 | 18.90 | 35 | 28.46 | 26 | 20.80 |  |
| Mixed | 44 | 35.20 | 40 | 31.75 | 46 | 36.22 | 35 | 28.46 | 39 | 31.20 |  |
| No religion /traditionalist | 24 | 19.20 | 31 | 24.60 | 38 | 29.92 | 31 | 25.20 | 35 | 28.00 |  |
| Ethnic group: Luo | 119 | 95.20 | 118 | 93.65 | 118 | 92.91 | 116 | 94.31 | 115 | 92.00 | 0.866 |
| Other religion | 6 | 4.80 | 8 | 6.35 | 9 | 7.09 | 7 | 5.69 | 10 | 8.00 |  |
| Number of under-fives | 2 | (1, 2) | 2 | (1, 2) | 1 | (1, 2) | 1.6 | (1, 2) | 1 | (1, 2) | 0.095 |
| Household size | 6 | (4, 7) | 6 | (5, 7) | 5 | (4, 7) | 5.6 | (4, 6) | 5 | (4, 6) | 0.055 |
| Energy intake (kcal/d) | 886 | (710, 1054) | 909 | (553, 1335) | 970 | (552, 1333) | 1139 | (847, 1465) | 1073 | (867, 1361) | <.0001 |

Data presented as medians and interquartile ranges (IQR) for continuous variables and as counts (proportions, %) for categorical variables. P values for categorical variables were calculated by x ^2^ test.

**Supplementary Table 6: Socio-demographic characteristics and food group intakes across quintiles of the ‘Pattern 3’**

| **Characteristics** | **Q1 (n=125)** | | **Q2 (n=125)** | | **Q3 (n=125)** | | **Q4 (n=126)** | | **Q5 (n=125)** | | **P for trend** |
| --- | --- | --- | --- | --- | --- | --- | --- | --- | --- | --- | --- |
| Male sex | 70 | 56.00 | 63 | 50.40 | 59 | 47.20 | 75 | 59.52 | 72 | 57.60 | 0.251 |
| Child’s age (months) | 10 | (7, 16) | 14 | (10, 19) | 15 | (11, 19) | 16 | (13, 20) | 17 | (13, 20) | <.0001 |
| Mother's age (years) | 29.2 | (24, 34) | 29.2 | (25, 34) | 28 | (23, 33) | 29 | (29, 34) | 29 | (24, 35) | 0.598 |
| Elementary education | 87 | 69.60 | 84 | 67.20 | 81 | 64.80 | 82 | 65.08 | 80 | 64.00 | 0.886 |
| Occupation: Housewife | 18 | 14.40 | 21 | 16.80 | 26 | 20.80 | 26 | 20.63 | 27 | 21.60 | 0.763 |
| Farmer | 68 | 54.40 | 76 | 60.80 | 66 | 52.80 | 66 | 52.38 | 63 | 50.40 |  |
| Skilled labourer | 32 | 25.60 | 21 | 16.80 | 23 | 18.40 | 25 | 19.84 | 24 | 19.20 |  |
| Business | 7 | 5.60 | 7 | 5.60 | 10 | 8.00 | 9 | 7.14 | 11 | 8.80 |  |
| Marital status: married | 100 | 80.00 | 98 | 78.40 | 92 | 73.60 | 103 | 81.75 | 91 | 72.80 | 0.349 |
| Religion: Protestants | 22 | 17.60 | 22 | 17.60 | 22 | 17.60 | 27 | 21.43 | 18 | 14.40 | 0.256 |
| Catholic | 25 | 20.00 | 34 | 27.20 | 26 | 20.80 | 38 | 30.16 | 29 | 23.20 |  |
| Mixed | 38 | 30.40 | 37 | 29.60 | 50 | 40.00 | 38 | 30.16 | 41 | 32.80 |  |
| No religion /traditionalist | 40 | 32.00 | 32 | 25.60 | 27 | 21.60 | 23 | 18.25 | 37 | 29.60 |  |
| Ethnic group: Luo | 116 | 92.80 | 108 | 86.40 | 119 | 95.20 | 121 | 96.03 | 122 | 97.60 | 0.003 |
| Other religion | 9 | 7.20 | 17 | 13.60 | 6 | 4.80 | 5 | 3.97 | 3 | 2.40 |  |
| Number of under-fives | 2 | (1, 2) | 2 | (1, 2) | 2 | (1, 2) | 1 | (1, 2) | 1 | (1, 2) | 0.444 |
| Household size | 6 | (4, 7) | 5 | (4, 6) | 5 | (5, 7) | 5 | (4, 6) | 6 | (5, 7) | 0.012 |
| Energy intake (kcal/d) | 681 | (422, 1076) | 875 | (589, 1132) | 903 | (655, 1178) | 1143 | (899, 1455) | 1205 | (1049, 1459) | <.0001 |

Data presented as medians and interquartile ranges (IQR) for continuous variables and as counts (proportions, %) for categorical variables. P values for categorical variables were calculated by x ^2^ test.

**Supplementary Table 7:** **Rotated factor loadings of three dietary patterns among breastfed children in Siaya County (n = 463)**

| **Food Category** | **Factor 1** | **Factor 2** | **Factor 3** |
| --- | --- | --- | --- |
| Fish | **0.70** | -0.08 | -0.22 |
| Vegetables | **0.68** | 0.07 | 0.27 |
| Fermented foods (ugali) | **0.59** | -0.13 | -0.36 |
| Rice and Pasta | **0.57** | 0.13 | 0.07 |
| Legumes | **0.55** | -0.08 | 0.17 |
| Fruit | **0.54** | 0.16 | -0.10 |
| Potatoes | **0.50** | 0.39 | -0.09 |
| Red meat | **0.50** | 0.04 | 0.15 |
| Poultry | **0.49** | 0.05 | -0.06 |
| Egg | **0.46** | 0.11 | 0.11 |
| Other oils | 0.41 | 0.25 | 0.09 |
| Coffee and tea | 0.37 | -0.13 | 0.05 |
| Vegetable soups, stews and sauces | 0.35 | -0.23 | 0.18 |
| Cakes and Sweets | 0.34 | 0.05 | -0.06 |
| Condiments | 0.32 | 0.06 | 0.07 |
| Sweet spreads | -0.02 | **0.70** | -0.01 |
| Vegetarian mixed dishes | -0.07 | **0.65** | -0.06 |
| Sodas and Juices | 0.07 | **0.64** | 0.09 |
| Dairy products | 0.01 | **0.45** | 0.02 |
| Processed meat | 0.06 | **0.45** | 0.19 |
| Roots and tubers | 0.22 | 0.33 | 0.0 |
| Margarine | 0.23 | 0.12 | **0.50** |
| Whole grain breads and cereals | -0.03 | 0.12 | **0.49** |
| White bread and cereals | 0.25 | 0.12 | **0.49** |
| Olive oil | -0.06 | -0.07 | **0.45** |
| Palm oil | 0.09 | 0.0 | **0.42** |
| Cooking fats | 0.05 | -0.06 | **0.42** |
| Nuts and seeds | 0.15 | 0.15 | 0.36 |
| Meaty mixed dishes | -0.11 | -0.01 | -0.02 |
| Eigenvalues percentage | 4.04 | 2.27 | 2.05 |
| Eigenvalues percentage contribution (% explained variance) | **13.9** | **7.8** | **7.1** |
| Total explained variance by all patterns | **28.8%** | | |

***Food groups with ≥ |0.40| factor loadings were considered major contributors to the pattern scores.***

**Supplementary Table 8: Subgroup analysis for the associations of three dietary pattern scores with the status of climate-sensitive micronutrients among breastfed children aged 6-23 in Siaya County (n = 463)**

| **Outcome** | **n (%)** | **Crude Model** | | **Model 3** | |
| --- | --- | --- | --- | --- | --- |
|  |  | **OR (95% CI)** | **p-value** | **OR (95% CI)** | **p-value** |
| **Pattern 1** | | | | | |
| Zn inadequacy | 302 (65.2) | 0.11 (0.07, 0.16) | <.0001 | 0.46 (0.26, 0.82) | 0.008 |
| Fe inadequacy | 404 (87.3) | 0.10 (0.06, 0.18) | <.0001 | 0.29 (0.14, 0.61) | 0.001 |
| Se inadequacy | 58 (12.5) | 0.16 (0.10, 0.26) | <.0001 | 0.28 (0.11, 0.69) | 0.006 |
| Vit A inadequacy | 159 (34.3) | 0.14 (0.11, 0.16) | <.0001 | 0.34 (0.19, 0.62) | 0.000 |
| MAR < 80% | 253 (54.6) | 0.09 (0.06, 0.13) | <.0001 | 0.18 (0.08, 0.39) | <.0001 |
| **Pattern 2** | | | | | |
| Zn inadequacy | 302 (65.2) | 0.75 (0.61, 0.93) | 0.009 | 0.80 (0.56, 1.14) | 0.216 |
| Fe inadequacy | 404 (87.3) | 0.99 (0.73, 1.35) | 0.965 | 1.61 (0.96, 2.70) | 0.071 |
| Se inadequacy | 58 (12.5) | 0.91 (0.66, 1.26) | 0.571 | 1.08 (0.60, 1.94) | 0.792 |
| Vit A inadequacy | 159 (34.3) | 0.47 (0.35, 0.63) | <.0001 | 0.43 (0.27, 0.68) | 0.000 |
| MAR < 80% | 253 (54.6) | 0.68 (0.55, 0.85) | 0.001 | 0.91 (0.56, 1.48) | 0.698 |
| **Pattern 3** | | | | | |
| Zn inadequacy | 302 (65.2) | 0.76 (0.62, 0.94) | 0.011 | 0.83 (1.34, 2.48) | 0.0001 |
| Fe inadequacy | 404 (87.3) | 0.55 (0.42, 0.72) | <.0001 | 0.99 (0.72,1.36) | 0.931 |
| Se inadequacy | 58 (12.5) | 0.87 (0.63, 1.21) | 0.406 | 2.57 (1.49, 4.42) | 0.001 |
| Vit A inadequacy | 159 (34.3) | 0.49 (0.38, 0.66) | <.0001 | 0.48 (0.27, 0.85) | 0.012 |
| MAR < 80% | 253 (54.6) | 0.69 (0.55, 0.86) | 0.001 | 2.41 (1.59, 3.65) | <.0001 |

Logistic regression was used to calculate odds ratios (OR), 95% confidence intervals (CI), and p-values.

Zn = zinc, Fe = iron, Se = selenium, Vit A = vitamin A, MAR = Mean Adequacy Ratio.

Crude: no adjustments

Model 3: Crude model adjusted for child's sex and age; maternal age, maternal education, maternal occupation, maternal marital status, number of under-fives, household size; energy intake (kcal/d), dietary fibre (g/d).
